# Navigating the Microbial Landscape: Understanding Dysbiosis in Human Genital Tracts and Its Impact on Fertility

**DOI:** 10.7759/cureus.67040

**Published:** 2024-08-16

**Authors:** Prachi A Ughade, Deepti Shrivastava, Kamlesh Chaudhari

**Affiliations:** 1 Obstetrics and Gynaecology, Jawaharlal Nehru Medical College, Datta Meghe Institute of Higher Education and Research, Wardha, IND

**Keywords:** sperm quality, bacterial vaginosis, reproductive health, fertility, genital microbiota, dysbiosis

## Abstract

Dysbiosis, an imbalance in microbial communities, significantly impacts the health and functionality of the human genital tract, with profound implications for fertility and reproductive health. This review explores the intricate relationship between genital tract microbiota and reproductive outcomes, highlighting the composition and dynamics of these microbial communities in both females and males. In females, the vaginal microbiota, primarily dominated by Lactobacillus species, is essential for maintaining a healthy vaginal environment, preventing infections, and supporting reproductive functions. In males, the genital microbiota influences sperm quality and reproductive health. Dysbiosis in the genital tract, manifesting as bacterial vaginosis, yeast infections, urethritis, or prostatitis, disrupts these microbial communities, leading to adverse reproductive outcomes such as infertility, pregnancy, and increased susceptibility to sexually transmitted infections. This review delves into the mechanisms through which dysbiosis affects fertility, including alterations in vaginal pH, mucosal immunity, inflammation, sperm viability, and motility. It also evaluates diagnostic methods, clinical implications, and management strategies, including probiotics, prebiotics, antibiotics, antifungal treatments, lifestyle interventions, and emerging therapeutic approaches. By understanding the microbial landscape of the genital tract and its impact on fertility, this review aims to inform targeted interventions that restore microbial balance and enhance reproductive health, ultimately improving fertility outcomes and the potential for healthy pregnancies.

## Introduction and background

Dysbiosis refers to an imbalance in the microbial communities inhabiting a specific environment, disrupting normal physiological functions [1]. In the context of the human genital tract, dysbiosis can manifest as shifts in microbial composition, potentially altering local immune responses and metabolic activities. This imbalance can result from various factors, including antibiotic use, hormonal changes, and lifestyle factors, ultimately affecting the health and function of the genital tract [1]. The human genital tract harbors diverse microbial communities that vary between males and females. In females, the vaginal microbiota is predominantly composed of Lactobacillus species, which play a crucial role in maintaining vaginal health by producing lactic acid, thus creating an acidic environment that inhibits the growth of pathogenic bacteria [2]. The composition of the vaginal microbiota can fluctuate due to factors such as menstrual cycle, sexual activity, and hormonal changes. In males, the genital microbiota includes various bacteria that can influence sperm health and reproductive outcomes. The balance of these microbial communities is essential for maintaining the health of the genital tract and supporting reproductive functions [3].

The composition and stability of the genital microbiota are increasingly recognized as significant factors in fertility and reproductive health. Dysbiosis in the female genital tract, such as bacterial vaginosis or fungal infections, has been linked to infertility, adverse pregnancy outcomes, and an increased susceptibility to sexually transmitted infections [4]. These microbial imbalances can disrupt the vaginal environment, impair sperm viability, and affect the immune response, all of which are critical for successful conception and pregnancy. In males, dysbiosis may negatively impact sperm quality, leading to reduced motility, altered morphology, and decreased fertility. Understanding the role of the genital microbiota in reproductive health is crucial for identifying potential interventions to restore microbial balance and improve fertility outcomes [5].

This review explores the impact of dysbiosis in the human genital tract on fertility and reproductive health. Specific objectives include examining the composition and dynamics of microbial communities in the female and male genital tracts and investigating the mechanisms through which dysbiosis influences fertility, including effects on sperm health, hormonal balance, and reproductive organ function. Additionally, the review will evaluate diagnostic methods and clinical implications of genital dysbiosis, discussing current and emerging strategies for managing dysbiosis to improve fertility outcomes. By understanding these aspects, we can develop targeted interventions that promote microbiome balance and enhance reproductive health, ultimately improving the chances of conception and healthy pregnancies.

## Review

The human genital microbiome

Composition of the Vaginal Microbiome

The vaginal microbiome is a crucial element of female reproductive health, characterized by a diverse array of microorganisms. Understanding its composition and the role of dominant bacterial species, particularly Lactobacillus, is essential for maintaining vaginal health and preventing disorders [6]. A healthy vaginal microbiome is primarily composed of Lactobacillus species, which are vital for sustaining a balanced microbial environment. The predominant Lactobacillus species in the vagina include Lactobacillus crispatus, Lactobacillus jensenii, Lactobacillus gasseri, and Lactobacillus iners. Collectively, these species contribute to a low-diversity microbiome that is essential for protecting against infections and supporting overall vaginal health. In contrast, conditions such as bacterial vaginosis (BV) are marked by a significant reduction in Lactobacillus and an increase in other anaerobic bacteria, such as Gardnerella vaginalis. This imbalance can lead to adverse health outcomes, including sexually transmitted infections and complications during pregnancy [7]. Lactobacillus species play several critical roles in the vaginal microbiome. They metabolize glycogen to produce lactic acid, which lowers the vaginal pH. A lower pH creates an environment that is hostile to pathogenic bacteria, thus providing a protective barrier against infections. Additionally, these bacteria produce various antimicrobial compounds, including hydrogen peroxide and bacteriocins, which inhibit the growth of harmful microorganisms [8]. Lactobacillus species also help modulate the local immune response, fostering a balanced immune environment that can effectively combat pathogens without triggering excessive inflammation. By maintaining dominance in the vaginal microbiome, Lactobacillus species help preserve a stable microbial community, preventing the overgrowth of opportunistic pathogens and reducing the risk of dysbiosis [7].

Composition of the Male Genital Microbiome

The male genital microbiome is a complex ecosystem composed of diverse bacterial species that significantly influence reproductive health. Key bacterial genera commonly found in the male genital tract include Corynebacterium, Staphylococcus, Lactobacillus, Prevotella, and various anaerobic Gram-positive cocci [9]. Corynebacterium species are typically present as commensals but can occasionally be associated with conditions such as prostatitis and urethritis. Staphylococcus epidermidis is among the most frequently detected bacteria in the male genital tract, while Lactobacillus species, though less prevalent than in the female genital microbiome, are also present. The interaction of these bacteria reflects a complex balance that can impact overall reproductive health [10]. The composition of the male genital microbiome profoundly affects sperm quality and fertility. Dysbiosis, an imbalance in microbial communities, has been linked to various reproductive issues. Studies have demonstrated that certain bacteria present in seminal fluid can negatively impact sperm motility and overall sperm parameters [11]. For example, an overgrowth of specific pathogenic bacteria may cause inflammation, impairing sperm function and reducing the likelihood of successful fertilization. Dysbiosis can also lead to oxidative stress and apoptosis in spermatozoa, compromising reproductive potential [12]. Understanding the dynamics of the male genital microbiome is essential for addressing infertility and developing targeted interventions. By identifying bacterial populations that contribute to either healthy or impaired reproductive outcomes, healthcare providers can explore personalized strategies to improve fertility. These approaches may include probiotics or other microbiome-modulating therapies to restore a balanced microbial environment in the male genital tract. As research in this field progresses, it offers promising opportunities for enhancing reproductive health and addressing male infertility more effectively [5].

Factors Influencing Genital Microbiota

**Figure 1:**
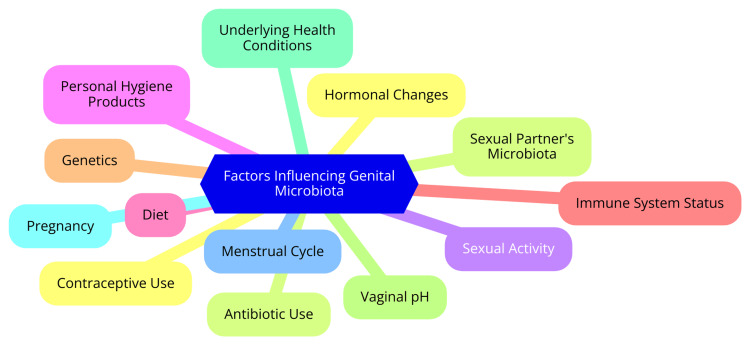
Factors influencing genital microbiota. Image credit: Prachi A. Ughade.

Several factors significantly influence the composition and stability of genital microbiota, affecting reproductive health. Understanding these factors is essential for maintaining a healthy microbial environment in the genital tract [3]. Hormonal fluctuations, particularly in estrogen and progesterone levels, play a critical role in shaping the vaginal microbiome. Research indicates that microbial diversity and the abundance of Lactobacillus species vary throughout the menstrual cycle. For example, increased microbial diversity is often observed during menstruation, while higher levels of Lactobacillus are associated with the follicular phase [13]. Hormonal contraceptives can alter or stabilize the microbiome's composition by influencing hormonal levels. The relationship between hormones and microbiota is complex; elevated estrogen levels can promote the growth of beneficial bacteria, whereas hormonal imbalances may lead to dysbiosis [14]. Sexual practices significantly impact genital microbiota as well. The exchange of bacteria between partners during unprotected intercourse can lead to changes in each partner's microbiota composition. Studies have shown that sexual practices, including the type of sexual partners and frequency of intercourse, can affect the diversity and stability of the vaginal microbiome. Women who have sexual contact with other women may exhibit different microbial profiles compared to those with male partners. This dynamic underscores the importance of considering sexual behavior when evaluating the health of the genital microbiome [15]. Hygiene practices are another critical factor that can disrupt the natural balance of the vaginal microbiome. Using soaps, douches, and other cleansing products can inadvertently lead to dysbiosis, marked by a decrease in beneficial bacteria and an increase in potentially harmful microorganisms. Overwashing or using harsh products can compromise the delicate microbial ecosystem. Therefore, maintaining a balanced hygiene routine is essential for preserving a healthy microbial environment in the genital tract. Gentle cleansing methods are generally recommended to support microbial health [16]. Finally, antibiotic use can dramatically alter genital microbiota by indiscriminately killing harmful and beneficial bacteria. This disruption can lead to dysbiosis, increasing the risk of infections and adversely affecting reproductive health. Studies have shown that antimicrobial drugs can significantly impact the composition of the genitourinary microbiota, particularly in vulnerable populations such as the elderly. The consequences of antibiotic-induced dysbiosis highlight the need for judicious antibiotic use and strategies to restore a healthy microbiome following treatment [17]. Factors influencing genital microbiota are shown in Figure 1.

Dysbiosis in the genital tract

Definition and Types of Dysbiosis

Dysbiosis refers to an imbalance in the microbial communities within the body, particularly in the gut and genital tract. This condition can lead to various health issues, including infections and fertility problems. Below are specific types of dysbiosis relevant to the genital tract [18]. Bacterial vaginosis (BV) is characterized by a disruption in the vaginal microbiota, where the normally dominant Lactobacillus species are reduced, allowing other bacteria, such as Gardnerella vaginalis, to proliferate [19]. This imbalance may cause symptoms such as thin, grayish vaginal discharge with a fishy odor, itching, irritation in the vaginal area, and an increased risk of infections. BV can also predispose individuals to sexually transmitted infections (STIs) and complications during pregnancy. It is considered a form of dysbiosis because it disrupts the vagina's normal protective flora, leading to various health complications if untreated [19]. Vaginal yeast infections, primarily caused by an overgrowth of Candida species, represent another type of dysbiosis. Contributing factors include antibiotic use, which can disrupt the normal flora and allow Candida to thrive; hormonal changes during menstruation or pregnancy that promote yeast growth; and dietary and lifestyle factors, such as high sugar intake and stress [20]. Symptoms of a yeast infection include itching and burning, especially during urination or intercourse, thick, white discharge resembling cottage cheese, and redness and swelling in the vaginal area. This type of dysbiosis highlights the delicate balance of microbial populations in the genital tract, where an overgrowth of yeast can cause significant discomfort and health issues [21]. In men, dysbiosis can manifest as urethritis and prostatitis, conditions characterized by inflammation of the urethra and prostate, respectively. These conditions may result from a bacterial imbalance, where pathogenic bacteria overgrow and disrupt the normal urogenital flora, or from sexually transmitted infections, which can lead to urethritis. This underscores the role of dysbiosis in infectious processes [22]. Symptoms may include painful urination with a burning sensation, unusual discharge from the penis, and pelvic pain in the lower abdomen or perineum. These conditions emphasize the importance of maintaining a healthy microbial balance for overall urogenital health and fertility in men [23].

Diagnostic Methods for Dysbiosis

Dysbiosis, an imbalance in microbial communities, can be diagnosed using various methods, including clinical assessments, microbiological assays, and molecular techniques. Understanding these diagnostic approaches is crucial for identifying dysbiosis and evaluating its potential health impacts [24]. The diagnostic process typically begins with thoroughly evaluating clinical signs and symptoms. Common indicators of dysbiosis include gastrointestinal symptoms such as bloating, diarrhea, constipation, and abdominal discomfort, which are often associated with disturbances in the gut microbiome. In women, genitourinary symptoms such as unusual vaginal discharge, itching, or burning may suggest dysbiosis, while men might experience similar symptoms in the penile area [25]. Oral dysbiosis can manifest as bad breath, gum disease, and tooth decay. Additionally, systemic symptoms such as fatigue, mood changes, and cognitive difficulties may arise if dysbiosis affects overall health. A detailed medical history and physical examination are essential for identifying potential causes and correlating symptoms with dysbiosis [26]. Microbiological assays are crucial for confirming dysbiosis through laboratory testing. The Comprehensive Digestive Stool Analysis (CDSA) is a common method that examines stool samples to identify and evaluate the balance of various bacteria, yeasts, and fungi in the gut, offering insights into microbial diversity and potential dysbiotic states. The Organic Acids Test, which measures organic acids produced by bacteria in urine, can reveal specific bacterial imbalances through abnormal levels [27]. The Hydrogen Breath Test is particularly effective for diagnosing small intestinal bacterial overgrowth (SIBO); it involves consuming a sugar solution and analyzing breath samples for hydrogen or methane gases produced by bacteria, helping to identify gastrointestinal dysbiosis [28]. In recent years, molecular techniques have gained prominence for providing a detailed understanding of dysbiosis. DNA sequencing methods, such as 16S rRNA gene sequencing, allow for identifying and quantifying microbial species in a sample, offering a comprehensive view of the microbiome's composition and diversity [29]. Metagenomic analysis further enhances this by examining the genetic material from microbial communities to assess their functional capabilities and interactions, shedding light on how dysbiosis may contribute to disease processes. For cases involving infections or localized dysbiosis, biopsy analysis may be performed to enable histological examination of tissue samples, helping to identify specific pathogens or assess the microbiome's state [30].

Mechanisms linking dysbiosis to fertility issues

Impact on Female Fertility

Dysbiosis in the female genital microbiota can significantly affect fertility through various mechanisms, including altered vaginal pH, impacts on sperm viability and motility, inflammation that disrupts endometrial receptivity, and associations with pelvic inflammatory disease (PID) [31]. The vaginal microbiome is essential for maintaining an optimal pH level, typically between 4.0 and 4.5, during reproductive years. This acidic environment is crucial for defending against pathogens and supporting sperm viability. Dysbiosis can lead to an elevated vaginal pH, which adversely affects sperm motility and overall fertility. When the pH exceeds 6.0, sperm motility decreases, making it more difficult for sperm to reach and fertilize the egg [32]. Sperm thrive in a slightly alkaline environment, and dysbiosis can create conditions detrimental to sperm survival. An acidic vaginal environment, worsened by dysbiosis, can lead to reduced sperm motility and viability, thereby decreasing the likelihood of successful fertilization. The research underscores the importance of a balanced vaginal microbiome in optimizing conditions for sperm survival and function [33]. Dysbiosis can also trigger inflammatory responses that impair endometrial receptivity. Chronic inflammation of the endometrium can hinder implantation and the maintenance of pregnancy. Pathogenic bacteria associated with dysbiosis can cause persistent inflammation, disrupting the uterine lining's ability to support an embryo. This inflammation can interfere with hormonal balance and negatively impact fertility outcomes [34]. Moreover, dysbiosis is linked to an increased risk of PID, which can result from infections in the reproductive tract. PID may lead to scarring and damage to the fallopian tubes, contributing to infertility. Pathogenic bacteria related to dysbiosis can heighten the risk of developing PID, further complicating reproductive health and fertility prospects [35].

Impact on Male Fertility

The impact of dysbiosis on male fertility is complex, affecting sperm quality and function, inducing genital tract inflammation and oxidative stress, and leading to reduced semen parameters [36]. One of the most notable effects of dysbiosis is its influence on sperm quality and function. An imbalance in the microbial composition of the male reproductive tract can impair sperm motility and viability. Pathogenic bacteria in the seminal fluid may disrupt normal sperm function, negatively affecting fertility outcomes. Research indicates that alterations in the seminal fluid microbiome, particularly with increased pathogenic bacteria, are associated with conditions such as azoospermia (absence of sperm) and diminished sperm quality. This microbial disruption can hinder sperm's ability to reach and fertilize the egg, impacting male fertility [5]. In addition to affecting sperm quality, dysbiosis can induce genital tract inflammation and oxidative stress, both of which are detrimental to reproductive health. Bacterial infections in the male reproductive tract often trigger an inflammatory response, compromising sperm production and function [5]. This inflammation is frequently accompanied by oxidative stress, an imbalance between reactive oxygen species (ROS) and antioxidants. Elevated ROS levels can cause oxidative damage to sperm DNA and membranes, compromising sperm quality and fertility. The interaction between inflammation and oxidative stress creates a challenging environment for sperm health, making successful conception more difficult for affected men [37]. Furthermore, dysbiosis is linked to various adverse changes in semen parameters, including reduced sperm concentration, motility, and abnormal morphology. These changes can result from both direct microbial effects and the inflammatory responses they provoke. Chronic inflammation, for example, can lead to conditions like ejaculatory duct obstruction, which diminishes sperm output and quality. Consequently, men with dysbiosis may face an increased risk of infertility due to these compromised semen parameters [38].

Clinical implications and management strategies

Probiotics and Prebiotics

Probiotics and prebiotics play a crucial role in maintaining and restoring a healthy microbiota, with significant implications for reproductive health. Their mechanisms of action are multifaceted and beneficial for both gut and genital tract health. Probiotics, which are live beneficial bacteria, contribute to microbiota health by enhancing the epithelial barrier, thus preventing the translocation of harmful pathogens into the bloodstream [39]. They compete with pathogenic microorganisms for adhesion sites on mucosal surfaces, inhibiting their growth. Additionally, probiotics produce antimicrobial substances, such as bacteriocins and organic acids, that suppress pathogenic bacteria. They also interact with host immune cells to modulate the immune system, promoting a balanced immune response essential for reproductive health [39]. Prebiotics, non-digestible food components that promote the growth of beneficial bacteria, also support microbiota health. They serve as a food source for probiotics, enhancing their survival and activity in the gastrointestinal tract. By fostering a healthy gut microbiome, prebiotics can indirectly benefit reproductive health by improving hormone metabolism, such as estrogen, which is crucial for ovulation and fertility. The interaction between the gut microbiome and reproductive health is vital, as gut dysbiosis can lead to systemic inflammation and hormonal imbalances that adversely affect fertility [39]. Clinical studies have demonstrated the efficacy of probiotics and prebiotics in restoring healthy microbiota. For example, probiotics have been shown to effectively manage conditions like bacterial vaginosis and other forms of dysbiosis in the female genital tract, which are associated with infertility. In men, seminal fluid dysbiosis, characterized by microbial imbalances, is linked to poor sperm quality and fertility issues. Research suggests that restoring a healthy microbial balance through probiotics can improve sperm parameters and enhance reproductive outcomes in assisted reproductive technologies [40]. Furthermore, restoring a healthy microbiota through these interventions can improve overall health. Both the gut and reproductive microbiomes are indicators of systemic health. Addressing dysbiosis with probiotics and prebiotics targets fertility issues and offers broader health benefits, including reduced inflammation and improved hormonal balance. This holistic approach to reproductive health highlights the importance of maintaining a balanced microbiome for optimal fertility outcomes in both men and women [41].

Antibiotic and Antifungal Treatments

Antibiotics are primarily prescribed to treat bacterial infections, varying from mild to severe. Common indications include respiratory infections like pneumonia and bronchitis, urinary tract infections (UTIs), skin infections such as cellulitis, and gastrointestinal infections, particularly those caused by Clostridium difficile. These medications work by inhibiting bacterial growth or killing bacteria, making them essential for managing serious infections. However, their use must be carefully managed, as inappropriate or excessive use can lead to adverse effects, especially on the microbiome [42]. Antifungal treatments combat fungal infections, particularly in immunocompromised patients, such as those undergoing chemotherapy for leukemia or lymphoma. Prophylactic antifungal treatment is often administered to prevent infections due to decreased immunity. Antifungals target specific fungal pathogens, helping to restore health in patients at high risk for opportunistic infections [43]. The use of antibiotics and antifungals can significantly disrupt the microbiome balance. Antibiotics, in particular, can cause a substantial decrease in microbial diversity in the gut, impacting both bacterial and fungal communities. While bacterial populations may gradually recover, the fungal community often shifts from a state of mutualism - where bacteria and fungi coexist beneficially - to one of competition. This shift can lead to the overgrowth of pathogenic fungi, such as Candida albicans, resulting in conditions like antibiotic-associated diarrhea and increased susceptibility to fungal infections [44]. Similarly, antifungal treatments can also notably impact the microbiome, though the specifics are less well understood. Antifungal agents can alter bacterial communities' composition and functional capacity, affecting various metabolic activities within the gut. This disruption can lead to a loss of beneficial bacteria and a decline in overall microbial diversity, which is crucial for maintaining gut health and supporting immune function [45].

Lifestyle and Behavioral Interventions

Maintaining a healthy genital tract microbiome is essential for optimal fertility in both men and women. Comprehensive sexual health education plays a vital role as a lifestyle intervention, equipping young people with the knowledge, skills, attitudes, and values necessary to safeguard their sexual and reproductive health. This education should encompass topics such as anatomy, puberty, relationships, consent, contraception, and sexually transmitted infections (STIs), tailored to various age groups. High-quality sexual education has been demonstrated to delay the onset of sexual activity, promote safer sex practices, and reduce risks of violence and abuse [46]. Proper genital hygiene is crucial for maintaining a balanced vaginal microbiome in women. This includes regular bathing and wearing clean, breathable underwear. It is advisable to avoid douching, scented products, and harsh soaps, which can disrupt vaginal pH levels. Men should also ensure cleanliness and dryness in the genital area and avoid tight-fitting underwear to prevent excessive moisture and bacterial overgrowth [16]. Practicing safe sex, undergoing regular STI testing, and promptly treating any infections can help prevent genital tract infections and dysbiosis. A healthy diet rich in fiber and probiotic foods can support a balanced gut microbiome, which, in turn, influences the genital microbiome. Stress management techniques, such as exercise, meditation, and social support, can help reduce inflammation and promote a healthier microbiome [47].

Future therapeutic approaches

Future therapeutic strategies for addressing dysbiosis in the human genital tracts and enhancing fertility include microbiome transplantation and personalized medicine approaches. Fecal microbiota transplantation (FMT) involves transferring fecal material from a healthy donor to a recipient to restore a balanced gut microbiome [48]. This technique has effectively treated recurrent Clostridioides difficile infections and inflammatory bowel diseases. The potential application of FMT in fertility treatments is being investigated, as restoring a healthy microbiome may improve reproductive outcomes by addressing dysbiosis in the gut and potentially influencing the genital microbiome [48]. In addition to FMT, probiotics, prebiotics, and dietary modifications are other methods to modulate the microbiome. These approaches aim to enhance beneficial microbial populations and suppress harmful ones, potentially improving fertility. Advances in microbiome research allow for identifying specific microbial signatures associated with health and disease, which can be used to tailor interventions based on individual microbiome profiles. Personalized medicine may incorporate artificial intelligence (AI) to analyze microbiome data in conjunction with clinical information, facilitating the development of customized treatment plans that target specific dysbiotic profiles [49]. Looking ahead, the development of targeted drugs to address microbial imbalances contributing to infertility may become a reality. By understanding the interactions between the microbiome and traditional treatments, healthcare providers can optimize therapeutic strategies for individual patients. In summary, integrating microbiome transplantation and personalized medicine is promising for addressing dysbiosis in the genital tracts and improving fertility outcomes. Ongoing research and clinical trials will be crucial for fully realizing the benefits of these innovative approaches [50].

## Conclusions

In conclusion, the intricate balance of the genital microbiota plays a crucial role in maintaining reproductive health and fertility in both males and females. Dysbiosis, characterized by an imbalance in microbial communities, can significantly impact fertility by altering the local immune response, affecting sperm viability, and disrupting hormonal and reproductive organ functions. Understanding the composition and dynamics of the genital microbiota and the mechanisms through which dysbiosis influences reproductive health is essential for developing effective diagnostic and therapeutic strategies. Current and emerging interventions, including probiotics, antibiotics, and lifestyle modifications, offer promising approaches to restoring microbial balance and improving fertility outcomes. Continued research in this field is vital for advancing our knowledge and developing targeted treatments that enhance reproductive health and support successful conception and pregnancy.
